# Sport-based youth development interventions in the United States: a systematic review

**DOI:** 10.1186/s12889-019-6387-z

**Published:** 2019-01-18

**Authors:** Meredith A. Whitley, William V. Massey, Martin Camiré, Mish Boutet, Amanda Borbee

**Affiliations:** 1 Adelphi University, 1 South Avenue, Woodruff Hall, Room 172, Garden City, NY 11530 USA; 2 Oregon State University, Milam Hall 118L, 2520 SW Campus Way, Corvallis, OR 97331 USA; 3 University of Ottawa, 125 University Private, Room 345, Otttawa, K1N 6N5 Canada; 4 University of Ottawa, 65 University Private, Morisset Hall, 309D, Ottawa, K1N 6N5 Canada

**Keywords:** Youth development, Physical activity, Program, Research, USA

## Abstract

**Background:**

The growing number of sport-based youth development interventions provide a potential avenue for integrating sport meaningfully into the U.S. public health agenda. However, efficacy and quality must be reliably established prior to widespread implementation.

**Methods:**

A comprehensive search of databases, peer-reviewed journals, published reviews, and both published and unpublished documents yielded 10,077 distinct records. Title and abstract screening, followed by full-text screening using 6 criteria, resulted in 56 distinct studies (coalescing into 10 sport-based youth development intervention types) included in the synthesis. These studies were then independently assessed and critically appraised.

**Results:**

Limited efficacy data were identified, with the quality of methods and evidence largely classified as weak. Processes likely to contribute to the outcomes of sport-based youth development interventions were identified (e.g., predictors of ongoing engagement, alignment between target population and intervention, intervention design), although more rigorous research is needed on these and other processes. Physical health outcomes were only studied in 3 of the 10 intervention types.

**Conclusions:**

The evidence base does not yet warrant wide-scale implementation of sport-based youth development interventions for public health goals within the U. S., although there is promising research that identifies areas for further exploration.

**Electronic supplementary material:**

The online version of this article (10.1186/s12889-019-6387-z) contains supplementary material, which is available to authorized users.

## Background

Despite widespread recognition of physical inactivity as a global public health issue [1], there continues to be a trend toward sedentary lifestyles, particularly for youth [2]. This includes the United States (U.S.), with 37.4% of 12–19 year olds classified as overweight and 20.9% of these youth identified as obese [3]. Consequently, the promotion of physical activity is a national health priority. Despite physical activity being an inherent feature of sport, which is immensely popular among youth in the U.S. (e.g., more than 44 million participants) [4], the role of sport in promoting physical activity is frequently marginalized in the public health agenda [5, 6].

The perception of sport as a competitive activity permeated by aggressive masculinity, expressions of violence, and a high tolerance for injury, may partially explain the lack of sport in public health discourse [7]. Others have argued that practices are filled with drills and a focus on strategy over activity [5, 8]. From this perspective, the public health agenda appears incompatible with the performance-oriented focus of sport [6], often positioned as an “afterthought in U.S. public health campaigns” ([5], p. 20). Moreover, competitive sport can be exclusionary [9], particularly when social inequalities (e.g., socioeconomic position) and neighborhood characteristics (e.g., social fragmentation, crime incidence) disproportionately affect low-income youth [10, 11].

A response to these concerns is the growing number of sport-based youth development interventions founded on principles of inclusion and participation [12]. These interventions expand their focus beyond physical health, promoting holistic development in physical, cognitive, affective, social, and lifestyle domains [13]. This emphasis broadens the traditional public health narrative that takes a deficit-reduction approach, instead embracing the notion that “problem-free is not fully prepared, and that fully prepared is not fully engaged” ([14], p. 25). Thus, sport-based youth development interventions involve youth in physical activities that intentionally foster developmental assets (e.g., values, commitment to learning, social competencies, positive identity) and surround youth with protective factors (e.g., support, relationships, experiences, resources, opportunities) that have been shown to be important facilitators of health for at-risk youth [15, 16]. This represents a paradigm shift in which public health efforts are embracing strength-based approaches in addition to problem-prevention activities.

Despite the potential for sport-based youth development to contribute to U.S. public health goals, evidence is needed to support the effectiveness of these interventions prior to wide-scale implementation. As Berg and colleagues pointed out, “all public health programs must guard against the idea that merely providing opportunities will get people more physically active” ([5], p. 22). This notion is particularly true for sport-based youth development interventions, as they have been criticized on account of theoretical [17] and methodological rigor [18]. As such, the purpose of the current study was to systematically review sport-based youth development interventions in the U.S. The primary research question was: What is the quality of evidence reported for sport-based youth development interventions in the U.S.? Two secondary research questions also guided the study: In youth populations, are sport-based youth development interventions effective for improving public health-related goals (i.e., physical, social, mental), and What are the processes that contribute to the outcomes of sport-based youth development interventions?

## Methods

**Fig. 1:**
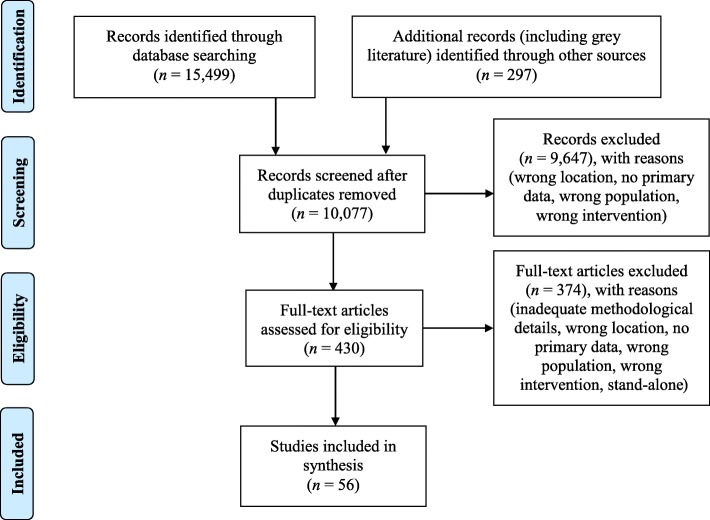
PRISMA flow diagram for research on sport-based youth development interventions in the U.S.

This systematic review was conducted and reported following the Preferred Reporting Items for Systematic Reviews and Meta-Analyses (PRISMA) guidelines (see Additional file 1 for PRISMA checklist) [19]. An electronic search was conducted of 6 databases (i.e., PsycINFO, Embase, SPORTDiscus, Education Source, Scopus, Web of Science; see Additional file 2 for detailed search terms and strategy), along with manual searches of 20 field relevant peer-reviewed journals and the reference lists of 12 recent reviews. Searches focused on literature published from 1995 through August 2017. Additionally, published and unpublished research documents were sought from 45 sport-based youth development experts, representing a diverse array of organizations, institutions, and groups, with the websites of these entities also searched. Overall, the search yielded 10,077 distinct records published in English (see Fig. 1), with the title and abstract of each record screened. Full text review of 430 articles (4.3%) was completed by 2 independent investigators using 6 screening criteria: (a) full text articles/evaluations with enough methodological data (i.e., a description of participants, data collection, and data analysis) to critically appraise the study; (b) data collected completely/partly in the U.S.; (c) empirical studies reporting primary data; (d) average participant age between 10 and 24 years old, per the United Nations’ definition of adolescents and youth [20]; (e) evidence of a plus-sport (i.e., sport adapted to maximize developmental objectives) or sport-plus (i.e., sport used as a vehicle for development, with precedence on non-sporting outcomes) intervention [21]; and (f) a minimum of 2 independent records for an intervention type (in response to consistent criticisms of one-off evaluations) [17, 22]. Disagreements were resolved through discussion with the primary investigator. The final sample contained 56 distinct studies, which coalesced into 10 intervention types: (a) Summer Sport and Life Skills Camps, (b) Teaching Personal and Social Responsibility, (c) Girls on the Run, (d) Playworks, (e) The First Tee, (f) Play It Smart, (g) Urban Squash, (h) Coach Across America, (i) Doc Wayne, and (j) Sport Hartford. A full reference list of all included studies is provided in Additional file 3.

### Quality assessment

Two investigators independently assessed and critically appraised the study methods. For studies containing quantitative data, the Quality Appraisal Tool for Quantitative Studies [23] was used, based on these criteria: (a) selection bias, (b) allocation bias, (c) control of confounding variables, (d) blinding, (e) data collection methods, (f) follow-up rates, (g) statistical analyses, and (h) intervention integrity (e.g., fidelity assessment, likelihood of co-intervention). Each study received a strong, moderate, or weak rating for each applicable criterion. When specific criteria were not applicable to a study (e.g., withdrawal and follow-up in a cross-sectional design; allocation bias in single group designs), the Quality Appraisal Tool for Quantitative Studies gives explicit instructions as to how to rate said criteria. An overall assessment was then derived: (a) strong if all criteria were rated moderate or strong; (b) moderate if 1 criterion was rated weak; and (c) weak if 2 or more criteria were rated weak. The investigators took a meta-theory and meta-method approach for qualitative studies [24]. A methodological coherence rating was given, which determined whether a study demonstrated consistency between ontology, epistemology, theory, methodology, sampling strategy, data collection techniques, data analysis, and study quality procedures.

### Data synthesis

Due to high levels of heterogeneity (see Table 1 for study details), a narrative description of the evidence is presented below. Specifically, the extant literature was examined within each intervention type, with the study outcomes assessed in the context of the quality of evidence. Where discrepancies across studies occurred (i.e., positive vs. negative results), these results are contextualized within the quality of the evidence.

**Table 1:** Summary table^1^

Intervention /Author (year)	Intervention Characteristics	Participants	Methodology	Analysis Strategy	Outcomes Addressed	Main Findings	Study Quality	Summary of Study Limitations
Summer Sport and Life Skills Camps								
Anderson-Butcher et al. (2013)	Participants attended a 4-week sport-based PYD summer camp for 5 h per day.	*N* = 193 Age 9–16 (Mean = 11.93)	Mixed methods, single group	Dependent *t*-test; content analysis of program observations	Social competence, sport-specific social competence, belonging	*Process:* Engagement and interaction between staff and youth noted as a major strength; behavioral management and social skill promotion were areas in need of improvement. *Efficacy:* No differences reported in social competence or belonging; pre-post improvements were reported in athletic competence (*p* < .01).	Weak; coherence between purpose and qualitative method. No philosophical assumptions discussed	Evidence of selection bias; no comparison group; large percentage of participants who did not complete measures; lack of blinded outcomes; less than optimal scale reliabilities; philosophical assumptions, data collection methods, and data analysis methods not reported in detail
Anderson-Butcher et al. (2014)	Participants attended a 4-week sport-based PYD summer camp for 5 h per day.	*N* = 287 Age 9–16 (Mean = 11.85)	Quantitative, single group	Latent growth curve modeling	Social competence, sport-specific competence, belonging, self-control, effort, teamwork, social responsibility	*Efficacy:* Social responsibility was reported to increase from pre-to-posttest. No other outcomes improved. Moderator analysis showed that those with lowest pre-test scores benefitted most from the program. Perceived belonging was shown to predict changes in outcome variables.	Weak	Evidence of selection bias; no comparison group; lack of reporting on withdrawals; lack of blinded outcomes
Gano-Overway et al. (2009)	See Newton et al.	*N* = 395 Age 9–16 (Mean = 11.8)	Quantitative, single group, cross-sectional	Test of mediation via structural equation modelling	Caring climate, emotional self-regulation, empathic self-efficacy, prosocial and antisocial behaviors	*Efficacy:* Affective self-regulatory efficacy and empathic self-efficacy mediated the relationship between a caring climate and youth’s social behaviors.	Weak	Cross-sectional study design; unable to account for likely covariates; amount of variance explained in models suggests other factors are salient in explaining behaviors
McDavid et al. (2015)	Participants attended a 4-week summer PYD through PA program for 6.5 h per day.	*N* = 321 Age 7–14 (Mean = 10.33)	Quantitative, single group	Latent variable longitudinal structural equation panel modeling	Self-worth, hope	*Efficacy:* No changes reported in self-worth or hope across the program. Gender and race did not moderate outcomes. Changes in self-worth predicted changes in hope, but only explained a small percent of the variance (2–7%).	Weak	Single group; non-causal design; measurement time lag; small effect sizes
McDavid et al. (2017)	Participants attended a 4-week summer PYD through PA program for 7 h per day. Group leaders were randomly assigned to receive standard training or training grounded in SDT.	*N* = 379 Age range NR	Quantitative, randomized control trial	Multi-level latent variable modeling, mediational analysis	Psychological need support, psychological need satisfaction, hope, self-worth	*Process:* Leader behavior was shown to influence child level outcomes (regardless of group). *Efficacy:* Changes in psychological need satisfaction predicted changes in hope and self-worth. Intervention did not affect leader behavior as intended.	Moderate	Lack of intervention effect; non-blinded outcomes
Newton et al. (2007)	Participants attended a 5-week summer camp sponsored by the NYSP. Camp sessions were attended daily and consisted of 4 h of PA, 1 hour of health education, lunch, and snacks. Groups were separated based on groups leader training. One group received a caring climate curriculum while other received the standard PYD curriculum.	*N* = 353 Age 9–17 (Mean = 12.18)	Quantitative, two-group, cross-sectional	Multivariate and univariate tests of covariance	Caring, perceived motivational climate, empathic concern, enjoyment, anticipated future participation	*Efficacy:* Caring group participants reported a higher level of perceived caring climate and lower level of perceived ego-oriented climate. Those in the caring group reported higher empathic concern and an increased likelihood for future involvement.	Weak	Evidence of selection bias; non-blinded measures; lack of control for confounding variables; no information reported on dropouts; post-test only design
Riciputi et al. (2016)	Participants attended a 4-week summer PYD through PA program for 6.5 h per day.	*N* = 24 Age 8–14	Qualitative, case study	Thematic analysis	Character, perceptions of program impact	*Process:* Program seen as a safe place where youth can build high-quality and reciprocal relationships. *Efficacy:* Participants discussed intrapersonal improvement (e.g., empowerment, values, behavior) and understanding of moral reasoning. Negative cases presented of three students not adhering to character concepts.	Philosophical underpinnings consistent with theory and method used in study	Use of grounded theory analysis techniques without completing a grounded theory study
Riley & Anderson-Butcher (2012)	Participants attended a 4-week summer PYD through PA program for 6 h per day.	*N* = 10 Age 31–58	Qualitative, general	Grounded theory approach	Camp outcomes for youth participants from parent perspective	*Efficacy:* Parents reported the camp provided general levels of biopsychosocial development, opportunities to explore broader horizons, and enhanced levels of psycho-social skills. Parents also reported having peace of mind knowing child was at program.	Lack of methodological coherence	Philosophical assumptions and specific methodology not reported; data analysis was a “grounded theory approach” but this was not consistent with study methodology
Riley et al. (2017; Riley, 2013)	Participants attended a 4-week sport-based PYD summer camp for 6 h per day.	*N* = 23 Staff *N* = 329 Youth Age 9–15 (youth)	Quantitative, single group pre-post	Multi-level modeling (youth outcomes nested within coach groups)	Social skills, youth-perceived staff practices	*Efficacy:* Self-control had a small (*d* = .29) but statistically significant increase from pre to post test. No statistical changes were noted in externalizing behaviors. Perceived emotional support was significantly related to perceived self-control (*b* = 1.13; *p* = .001); neither emotional support or autonomy support was predictive of reductions in externalizing behaviors.	Weak	Evidence of selection bias; lack of comparison group; non-blinded outcomes
Ullrich-French & McDonough (2013)	Participants attended a 4-week summer PYD through PA program for 6 h per day. Participants had to complete Year 1 of the summer PYD program and be eligible to come back in Year 2.	*N* = 215 Age 8–13 (Mean = 11.6)	Quantitative, single group, longitudinal	Logistic regression, multivariate analysis of covariance	Leader support, social competence, physical competence, self-worth, attraction to PA, hope	*Efficacy:* BMI (OR = 0.91), self-worth (OR = 2.15), program attendance (OR = 1.49), and perceptions of leader support (OR = 1.70) increased the likelihood that participants returned the following year.	Weak	Lack of data from those non-returners; lack of blinded outcome measures
Ullrich-French et al. (2012)	Participants attended a 4-week summer PYD through PA program for 6 h per day.	*N* = 197 Age 9–16 (Mean = 11.8)	Quantitative, single group	Repeated measures multivariate analysis of variance	Leader support, social competence, physical competence, self-worth, attraction to PA, hope	*Efficacy:* Perceived social competence, perceived physical competence, physical self-worth, and global self-worth increased from pre to post program; no other variables showed change. Results were moderated by age, with older children experiencing more benefit from the program.	Weak	Lack of control group; lack of blinded outcome measures; short intervention and follow-up period
Teaching Personal and Social Responsibility								
Cryan & Martinek (2017)	Intervention was an after-school soccer program grounded in TPSR principles. Participants attended the program 2 hours per day, 2 days per week, for 9 weeks.	*N* = 14 Age = 11–12	Mixed methods, single group pre-post	Deductive analysis; dependent *t*-test	Personal and social responsibility	*Efficacy:* There were no within group differences reported for personal responsibility, however within group improvements of social responsibility were noted. Qualitative data suggests that initiative and teacher-student relationships improved during the program. Teacher interviews suggest an increase in classroom behavior.	Weak; lack of coherence	Single group study; small sample size; selection bias; inconsistent attendance during intervention; non-blinded outcomes
Hayden et al. (2012; Hayden, 2010)	Intervention was a school-based program that used life skills programming and physical activity within the TPSR model. Students met twice per week for 1 hour over the course of the school year.	*N* = 63 Age = 9-12th grade	Qualitative, program evaluation	Content analysis; descriptive statistics	TPSR implementation, academics, social-emotional supports	*Process:* Data suggest that TPSR components of integration, transfer, empowerment, and teacher-student relationships were followed. *Efficacy:* Students perceived increased effort in the classroom, increased positive communication with teachers, increased desire for academic accountability, increased effort in sport, increased sense of belonging, a sense of being a role model, accountability to teammates, and more care from adults. Advisors reported increased effort in the classroom along with increased participation and social-emotional development in the sport context (leadership, empowerment, boundaries, and healthy risk-taking).	Coherence between theory, methods, and analysis. No philosophical assumptions discussed	Lack of female participants; lack of differentiation between researcher and advisor roles; disconnect between cultural norms of key stakeholders
Jacobs (2016a)	Intervention was school-based and conducted within a volleyball unit of a physical education curriculum using TPSR principles. The study lasted 15 days.	*N* = 122 Age = 11–14	Quantitative, quasi-experimental	Factorial analysis of variance	Youth experience, transfer of life skills	*Efficacy:* No group x time differences were reported for transfer of life skills. No effect on YES subscales for identity reflection, diverse peer relations, group processing skills, feedback leadership, and responsibility. Significant differences in youth experiences reported for: identity experiences (small effect .037), goal setting (small effect .043), effort (small effect .042), problem solving (moderate effect .108), time management (small effect, .06), emotional regulation (small effect .054), physical skills (moderate effect .102), prosocial norms (moderate effect .068); no effect on YES subscales for identity reflection, diverse peer relations, group processing skills, feedback leadership, and responsibility.	Weak	Lack of control for possible confounding variables; non-blinded outcomes; high risk for type I statistical error given the amount of analyses
Jacobs (2016b)	This qualitative paper examined program experiences in a community-based sport organization.	*N* = 11 Ages 12–18	Qualitative, phenomeno-graphic	Deductive analysis	Perceptions of life skill transfer, youth cognitive processes	*Efficacy:* Participants discussed personal impact of sport program, social responsibility, life skill development, and situational insights.	Lack of methodological coherence	Short interviews; lack of consistent methodology; highly deductive coding procedure
Martinek et al. (2006)	TPSR-based program in which youth leaders create physical activity lessons that reinforce life skills. Length, duration, and intensity of intervention were not reported.	*N* = 4 Age 14–17	Qualitative, case study	Case description	Developmental stages of youth leadership	*Efficacy:* Stage 1 is composed of needs-based leadership, Stage 2 is composed of planning and teaching, Stage 3 is composed of reflective leadership, and Stage 4 is composed of compassionate leadership.	Lack of methodological coherence	Philosophical assumptions, sampling strategy, sample description, data analysis methods, and validity assessments not reported
Melendez & Martinek (2015)	Intervention used sport clubs grounded in TPSR principles, mentoring, and youth leadership training. Intervention length, frequency, and intensity were not reported.	*N* = 5 Age = not reported	Qualitative, multiple case study	Deductive analysis	Program experiences	*Efficacy:* TPSR values deemed important to participants’ lives. Learned the value of helping others and leadership; however, the specific leadership program was not influential in teaching respect and caring values.	Philosophical assumptions, theory, and methods showed coherence	Small sample and lack of data regarding skills learned outside of program; deductive analysis
Miller (1997)	Intervention was school-based and conducted within a physical education course. Those in the intervention group participated in a TPSR-based socio-moral growth curriculum for 2 hours a day, 3 days a week, for 28 weeks.	*N* = 58 Age = 10–11	Quantitative, quasi-experimental	Analysis of co-variance	Distributive justice reasoning, perceived competence	*Efficacy:* Results showed significant differences when controlling for pre-test scores on DJR favoring the treatment group (*p* < .05). Examination of improvement rates showed an absolute benefit increase of 26.6%, a relative benefit increase of 83.13%, and an NNT of 4–5; differences were also reported between groups for behavioral PC (*p* < .004), but not for athletic competence; no differences were found for perceptions of task or ego climate between groups.	Strong	Floor and ceiling effects of the TEOSQ prevented meaningful analysis of that data
Schilling et al. (2007)	Intervention was a youth-led TPSR program that met 1 day per week throughout the school year and for 3 weeks in the summer.	*N* = 12 Age 13–18 (Mean = 16.7)	Qualitative, general	Inductive and deductive analysis	Youth perceptions of program and program commitment	*Process:* Antecedents to commitment: program environment, program structure, relationship, personal characteristics. Barriers to commitment: program logistics and structure, personal factors. Nature of commitment: behavior and emotional involvement.	Lack of methodological coherence	No specific philosophical or methodological underpinnings
Walsh (2008)	TPSR-based career club program in which sport is used to teach responsibility and older and younger participants are paired to work within a mentoring relationship. Program met once per week, for 90 min, across a 9-week timespan.	*N* = 12 7th and 8th grade	Qualitative, case study	Inductive analysis	Employment, education	*Efficacy:* Participants reported the importance of having to work hard and stay focused, increased communication skills, clarity about the future, determination, ability to see path to goals, and increased effort and performance in school.	Conflicting paradigms of subjectivity and objectivity noted	Perceptions of employment not tied to actual employment outcomes
Walsh et al. (2010)	TPSR-based coaching club intervention that included 45 sessions over two academic school years. Sessions were provided once a week for one hour.	*N* = 13 youth *N* = 3 adult leaders Age = 9–11	Qualitative, program evaluation	Inductive and deductive analysis	Transfer of TPSR goals	*Efficacy:* Transfer of respect to the school environment; transfer of self- and emotional control in the school yard; worked harder in school; took more ownership over action in school; helped others and learned how to be an example for others outside of the program.	Coherence between philosophical assumptions and sampling strategy	Authors discuss grounded theory analysis techniques, but did not conduct a grounded theory study
Walsh et al. (2012)	Kinesiology Career Club is a TPSR-based program aimed at helping high school youth explore future careers in kinesiology. Program met within a school setting, twice per week for 75 min over a 10- to 12-week period.	*N* = 14 Ages = 14–15	Qualitative, program evaluation	Inductive and deductive analysis	Impact of KCC	*Efficacy:* Results discussed helping participants connect TPSR goals to possible futures, envisioning and exploring a career in Kinesiology, and helping to balance hopes and fears.	Methodology, data collection, and data analysis showed coherence	Lack of philosophical underpinnings
Walsh et al. (2015)	Kinesiology Career Club is a TPSR-based program aimed at helping high school youth explore future careers in kinesiology. Program met within a school setting, twice per week for 75 min over a 10- to 12-week period.	*N* = 8	Qualitative, case study	Inductive and deductive analysis	Mentors perceptions of KCC	*Efficacy:* Results discussed positive perceptions of KCC goals and the ability of the program to transfer program goals to participants’ possible future selves.	Methodology, data collection, and data analysis showed coherence	Lack of philosophical underpinnings
Whitley et al. (2016)	TPSR-based program developed to address the challenges faced by refugee youth. Program met once per week for 60 min (number of weeks was not reported).	*N* = 16 Age 10–18	Qualitative, methodology not explicitly reported	Hierarchical content analysis	Program experiences	*Process:* Themes discussed included having fun, experiencing sports, being a member of a team, and developing a relationship with adults. *Efficacy:* Additional themes were learning sports, learning about respect, teamwork, and leadership, and transferring learning outside of program.	Lack of methodological coherence	Philosophical assumptions, methodology, and sampling strategy not explicitly addressed
Whitley et al. (2017)	TPSR model used to develop an 8-session program in collaboration between Southern Queens Park Association and Adelphi University. Visit to Adelphi University during 3rd and 10th week of programming to introduce participants to higher education, attend a class, dinner at campus cafeteria, meeting with admissions representative, and a sport event.	*N* = 7 youth participants Age = 11.86	Qualitative, community-based participatory research	Inductive and deductive analysis	Program implementation and youth development outcomes	*Process:* Program climate and leader/mentor strategies identified as key to outcomes. *Efficacy:* Skills learned, skills transferred, and intention to transfer personal and social responsibility, effort, self-regulation, leadership, empowerment, increase physical activity interest and experience, improved physical abilities.	Methodological coherence	Small sample size; limited program space; deductive nature of the analysis
Wright & Burton (2008)	Intervention was a school-based Tai Chi program grounded in TPSR principles and conducted within a physical education course. Program met twice per week for 50 min over a 10-week timeframe.	*N* = 23 Age = 14.8	Qualitative, program evaluation	Inductive and deductive analysis	Program characteristics	*Process:* Results discussed establishing a relevant curriculum, practicing life skills within program, seeing the potential to practice life skills outside of program, and creating a valued program.	Coherence between framework, methodology, and analysis	Lack of philosophical underpinnings to study; reliance on deductive coding
Wright et al. (2010)	Intervention was school-based and conducted within a physical education course. Those in the intervention group received a Tai Chi intervention grounded in TPSR principles for approximately 18 weeks.	*N* = 122 (sub-sample of 11 interviewed in a focus group) Age 14–18 (Mean = 14.8)	Mixed methods, quasi-experimental, program evaluation	Inductive and deductive analysis; descriptive	Grades, tardiness, absences, conduct	*Efficacy:* No significant group differences reported. Grades dropped for both groups, but slightly more for the control group; Absences increased in both groups. There was an increase in positive behavior and decrease in negative behavior reported in relation to the control group; however, these differences were not reported to be statistically significant. Qualitative data suggested youth perceived improvement in the TPSR levels.	Weak; framework, methodology, and methods were coherent. Data analysis limited the results	Selection bias; unclear blinding protocols; gender differences not explored; study restricted to one school; lack of control for known co-variates; deductive qualitative analysis
Wright et al. (2012)	TPSR-based Kung-Fu program that took place at a local YMCA. Program met once per week for 45–60 min across an academic school year.	*N* = 4 Age = 10–13	Qualitative, case study	Inductive and deductive analysis	Program experiences	*Process:* Overall positive program perceptions were discussed. *Efficacy:* Case profiles discuss lessons learned and skills developed for each participant.	Methodology, data collection, and data analysis showed coherence	Lack of philosophical underpinnings; lack of rich data
Girls on the Run								
Beller (2013)	Participants engaged in a 12-week program that meets twice per week for 1.5 h per session. The program combines training for a 5k with a positive youth development curricula. The curricula includes self-awareness and self-care (part 1), teambuilding, cooperation, and community building (part 2), and social contribution (part 3).	*N* = 209 High school-aged	Quantitative, case-control	Tests of group difference (t-test, ANOVA, chi-square)	Body image satisfaction, PA	*Efficacy:* No differences in body image satisfaction between those in GOTR and those not in GOTR; no differences in PA engagement either.	Weak	Self-reported PA data; selection bias; retrospective design; lack of control for potential confounding variable; non-blinded measures
Debate (2002)	See above.	*N* = 322 Age = 10	Quantitative, single group	Dependent *t-*test	Self-esteem, body image satisfaction, eating attitudes and behaviors	*Efficacy:* Significant improvements reported in self-esteem, body size satisfaction, and eating behaviors.	Weak	Single-group study; lack of control for confounding variables; self-reported outcomes
Debate & Delmar (2006)	See above.	*N* = 282 Age = 10.47	Quantitative, single group	Pre-post differences (*t*-test, Wilcoxon test)	Self-esteem, body image satisfaction, eating attitudes and behaviors, attitudes towards PA, empowerment, self-reported PA	*Efficacy:* Significant improvements reported in self-esteem, body size satisfaction, and PA behaviors, and some healthy eating and empowerment items.	Weak	Single-group study; lack of control for confounding variables; self-reported outcomes; different levels of exposure
Debate & Otero-Fisher (2005)	See above.	*N* = 157 Age = 10.25	Quantitative, single group pre-post	Pre-post differences (*t*-test, Wilcoxon test)	Self-esteem, body image satisfaction, eating attitudes and behaviors, attitudes towards PA, empowerment, self-reported PA	*Efficacy:* Significant improvements reported in self-esteem, body size satisfaction, and PA behaviors.	Weak	Single-group study; lack of control for confounding variables; self-reported outcomes
Debate et al. (2009)	See above.	*N* = 1034 Age 8–15	Quantitative, single group	Dependent *t-*test	Self-esteem, body size satisfaction, PA, PA commitment	*Efficacy:* Significant pre-post differences reported for self-esteem, body size satisfaction, and PA frequency.	Weak	Single-group study; large amounts of missing data; use of partial measures could question validity; unclear blinding protocols
Pettee Gabriel et al. (2011)	See above.	*N* = 877 Age 9–11	Quantitative, quasi-experimental	Repeated measures analysis of covariance	Self-esteem, body size satisfaction, PA, PA commitment	*Efficacy:* No group x time interaction effects were found for self-esteem, body size discrepancy, or PA commitment. Differences were noted in PA levels: those never exposed and newly exposed had greater change in PA scores at follow-up.	Weak	Selection bias due to low enrollment, non-blinded outcomes; data collected in one school district could confound results; possible seasonal differences in PA; inconsistent administration of survey data
Rauscher et al. (2013)	See above.	*N* = 138 Age 8–14 (Mean = 10.5)	Mixed methods, single group, formative evaluation	Inductive content analysis; dependent *t-*test	Body consciousness, body esteem, nutrition, self-efficacy, attitude toward PA and mentorship	*Process:* Participants and coaches were uncomfortable with body-conscious conversations, discrepancies between messages sent (need for “good body”) and program goals. *Efficacy:* Small but significant effects for pre-post body consciousness and body esteem. Healthy girl was defined as physically active, confident, good looking, thin, fit, strong, and nice.	Weak; lack of methodological coherence	Selection bias; lack of reporting on withdraws; non-blinded outcomes; qualitative data lacked depth
Waldron (2007)	See above.	*N* = 34 (sub-sample of 8 for qualitative interviews) Mean age = 11.51	Mixed methods, single group	Dependent *t*-test; grounded theory coding	Perceived competence, program experiences	*Efficacy:* Small but significant effects for pre-post perceived social competence, perceived physical competence, and perceived physical appearance competence. Qualitative data suggest increases in self-worth, social support, and perceived competence.	Weak; lack of methodological coherence	Single-group study; selection bias; non-blinded outcomes; small sample size; use of grounded theory coding, without grounded theory methodology
Playworks								
Beyler et al. (2013); Fortson et al. (2013); London et al. (2013)	See above.	*N* = 2331 student surveys; *N* = 296 teacher surveys; *N* = 1579 accelerometry data	Randomized controlled trial, cross-sectional analysis only (i.e., post-intervention comparison)	Multi-level regression models	Physical activity, school climate, student behavior	*Efficacy:* Children at Playworks schools had significantly higher levels of: (a) physical activity, (b) teacher-reported safety and inclusion, and (c) student-reported positive behavior and attention in class than those at control schools. Teachers also reported lower levels of bullying and transition difficulty. No differences were reported in youth development, children perceptions of safety, teacher-reported classroom behavior, or academic outcomes	Strong	Single time point measurement; limited accelerometry data
London et al. (2015)	See above.	*N* = 6 schools. Principal, recess coach, teacher interviews, recess observations at multiple time points, and student focus groups at each school	Qualitative, program evaluation	Grounded theory approach	Recess climate	*Efficacy:* Playworks implementation resulted in a higher quality recess. Higher-quality recess sessions contained higher levels of students initiating and sustaining games, higher levels of inclusion, higher levels of female participation, more positive language, less conflict and bullying, stronger connection to recess coach, and more teachers on the playground.	Partial methodological coherence; incongruence between study design and analysis techniques (i.e., grounded theory)	Lack of participant descriptions; unclear use of grounded theory methodology/analysis
Madsen et al. (2011)	School-based program in which full-time trained coaches work in schools and teach and coordinate a variety of playground sports and games; work with classroom teachers to provide additional PA opportunities; provide a peer leadership program; and work to generate family and community involvement.	*N* = 13,109 fifth-grade students	Quantitative, quasi-experimental, retrospective time series	Mixed effects linear regression	Internal and external assets as assessed by the California Healthy Kids Survey	*Efficacy:* With each additional year of exposure to Playworks, students reported significantly higher scores in PA, meaningful participation in school, problem solving skills, and goal aspirations; effects reported were small but clinically meaningful when considered across time and within the context of percentile rank.	Strong	Retrospective design; lack of control over data collection processes
Massey et al. (2017)	See above.	*N* = 450 children in observations; *N* = 21 children in classroom observations; *N* = 77 children in focus groups	Mixed methods, quasi-experimental, program evaluation	Repeated measures analysis of variance; factorial ANOVA; interpretive content analysis	Adult-student playground interactions, playground behavior, classroom behavior	*Process:* Playworks schools had significantly more positive adult-student interactions and significantly less conflict on the playground than a non-intervention comparison. *Efficacy:* Classroom data showed those in the peer leadership program improved their behavior relative to a control group.	Moderate, partial methodological coherence	Lack of comparison group at baseline for observations; non-randomized design; small sample for classroom observations; lack of detail on philosophical underpinnings and sampling strategy
Massey et al. (2018)	See above.	*N* = 77 Playworks Junior Coaches; *N* = 13 Playworks coaches	Qualitative, program evaluation	Interpretive content analysis	Leadership	*Process and Efficacy:* Participants discussed various aspects of leadership and how that influenced the decision to become a junior coach, the role of a junior coach, training received, and developmental impacts as a result of the experience.	Partial methodological coherence	Lack of detail on philosophical underpinnings and sampling strategy
The First Tee								
Brunelle et al. (2007)	Intervention was a condensed 1-week (5 sessions of 45 min each) version of The First Tee program that combines golf lessons with life skill development.	*N* = 100 Age 13–17	Quantitative, single group	Repeated measures analysis of covariance; regression analysis	Social responsibility, interpersonal reactivity, social interests, goals, community service	*Process:* Whether or not individuals completed their community service requirement had a significant effect on empathic concern and social responsibility. *Efficacy:* Authors noted significant pre-post (1 week) differences on social responsibility and goal knowledge. Gender and race were shown to moderate outcomes (girls showed greater increases in perspective-taking; being white was more predictive of social interests).	Weak	No true control group; self-report measures; short intervention time-frame; large percentage of loss to follow-up
Weiss et al. (2013)	Intervention consists of a program in which golf and life skills are taught in a systematic and progressive program that addresses interpersonal, self-management, goal setting, and advanced social skills. Program length, duration, or intensity was not reported.	*N* = 95 Age 11–17	Qualitative, interpretive	Inductive and deductive content analysis	Interpersonal and self-management skills, transfer of skills to other domains	*Efficacy:* Identified skill development in meeting and greeting others, showing respect, and emotion management within and outside of the program.	Methodological coherence from theory to method to analysis	Philosophical assumptions to study not addressed
Weiss et al. (2016)	See above.	N_1_ = 564 (405 in First Tee group) N_2_ = 192 (Longitudinal sample) Age 10–17	Quantitative, longitudinal, quasi-experimental	Multivariate analysis of covariance (group difference); latent growth modeling (intervention group only)		*Efficacy:* Data show significant group differences (when controlling for parent education and SES) on 5/8 life skill transfer domains and 6/8 developmental outcome domains. Longitudinal data showed that 3 life skills increased over time (with increased exposure to the program): meeting and greeting, appreciating diversity, and getting help. Those who entered the program with the lowest scores for life skills gained the most improvement over time.	Moderate	Non-blinded outcomes; unclear sampling procedures in Study 1; baseline differences in groups in Study 1
Play It Smart								
Petitpas et al. (2004)	Program is grounded in a life skills development framework and implemented through a coordinated effort of academic coaches (working 20 h per week to coordinate), parents, school personnel, and community leaders.	*N* = 252 Age 14–18	Quantitative, single group, longitudinal	Descriptive statistics	ACT/SAT scores, GPA, community service, self-reported health behaviors	*Efficacy:* Program participants saw in increase in GPA from 2.16 to 2.64. 98% of seniors in program graduated on time and 83% went to college. Participants engaged in 1745 h of community service.	Weak	Lack of methodological detail to judge the rigor of the data
Van Gorden et al. (2010)	See above.	*N* = 1361	Qualitative, general	Grounded theory coding	Life skills	*Efficacy:* Data from exit interviews showed that youth in the program perceived that they developed life schools, had academic and athletic accomplishments, engaged in community service, built relationships with important others, and had a more positive outlook on life.	Lack of methodological coherence	Data from exit interviews that were administered by academic coach; lack of philosophical or methodological underpinning
Urban Squash								
Green (2010)	Intervention is an academic sports mentoring program. Participants attend 3 days per week for 3 h each day (90 min of homework, 90 min of squash) across the school year.	*N* = 46 6th and 7th grade	Quantitative, quasi-experimental	Analysis of covariance	Intellectual functioning, academic functioning, academic achievement	*Efficacy:* No differences reported for academic engagement between groups. Intervention group showed significant improvements in reading and writing but not math. Intervention group reported significantly higher gains in GPA than control group. Achievement status did not moderate results.	Moderate	Small sample; high attrition rate; lack of power for number of analyses
Hemphill & Richards (2016)	See above.	*N* = 21 youth *N* = 13 staff Youth were in 6th – 8th grade	Qualitative, program evaluation	Descriptive statistics; grounded theory coding	Valued aspects of program, how outcomes may have transferred out of program	*Efficacy:* Results focused on academic enrichment, academic transfer, relationships, and a focus on personal and social responsibility.	Methodology, sampling and data collection consistent	Lack of philosophical underpinnings to study; use of grounded theory analysis without doing a grounded theory study
Hill (2012)	See above.	*N* = 111 Age = 11–14	Quantitative, quasi-experimental	Repeated measures analysis of variance	Intellectual functioning, academic functioning, academic achievement, social support	*Efficacy:* No differences found between groups on academic engagement, individual academic skills (math, oral language), or social support. Total academic achievement scale improved slightly for intervention group, but not control group.	Moderate	Lack of specificity in measurement; attrition rate; small sample
Coach Across America								
LPHI (2016a; 2016b)	Up2Us Sports is a national coalition of more than 1000 organizations committed to using sports for social change. Striving to harness the power of sports to reduce youth violence and promote health and academic success, Up2Us Sports organizes nationwide community training programs.	Approximately 1000 participants in older group and approximately 1100 in younger group	Mixed methods, quasi-experimental; qualitative general	Analysis of covariance; hierarchical linear regression modeling; content analysis	Fitness, high impact attributes (HIA), self-reported nutrition, coach quality, dose	*Process:* More time with coaches was related to outcomes, but coach quality was a negative predictor of HIA in younger children. *Efficacy:* Results showed program participants significantly improved their fitness level. Data on HIA and nutrition were mixed, with a greater effect for younger participants.	Moderate; lack of methodological coherence	Unclear information on missing data; lack of control for confounding variables
Windham et al. (2014)	See above.	*N* = 6288 *N* = 2229 pre-post surveys	Quantitative, single group	Hierarchical linear regression	Self-reported physical activity, self-reported nutrition behaviors, HIA	*Efficacy:* Significant effects reported for increased PA. Results on nutrition and HIA were mixed (improvements on 2/6 nutrition items; improvements on 2/8 HIA).	Weak	Large amounts of missing data; no information on reliability or validity of measures; unclear blinding procedures
Doc Wayne								
D’Andrea et al. (2013)	This intervention took place within a trauma treatment facility. Coaches were trained to deliver trauma-sensitive sports, and the sport program took place once per week, for 1 hour, over a 5-month period.	*N* = 88 Age = 12–21	Quantitative, quasi-experimental	Repeated measures analysis of variance	Behavior within the program, mental health	*Efficacy:* Significant effects for restraints, timeouts needed, internalizing behavior, and externalizing behavior were reported in favor of the treatment group.	Weak	Likely selection bias; unclear description of control for possible confounding variables; unclear blinding procedures; lack of reliability reported for observational measures
Program Evaluation Report (n.d.)	Trauma-informed sport league for youth in residential treatment. Timing, duration, and dosage not reported.	*N* = 53	Quantitative, single group	Pre-post effect sizes	Life goals, social conflicts, emotional regulation, behavior, stress, challenges	*Efficacy:* Compared to youth not in the sport league, those in the sport league reported less personal distress and more perspective taking, higher levels of emotional regulation, higher social cognition, and higher heart rate variability.	Weak	Likely selection bias; unclear description of control for possible confounding variables; unclear blinding procedures; lack of reliability reported for observational measures; missing data
Sport Hartford								
Bruening et al. (2015)	Mentor-based program that incorporated sport, physical activity, nutrition, and life skills. Sessions were twice a week for 2 hours over a 28-week period.	*N* = 5 Age = 12–15	Qualitative, general	Deductive analysis	Factors that influence program outcomes	*Process:* Active participation and planning, connection to community, sense of belonging, trust, information channels, norms, and sanctions.	Coherence between theory, method, and analysis	Lack of philosophical underpinnings
Bruening et al. (2009)	Mentor-based program that incorporated sport, physical activity, nutrition, and life skills. Sessions were once a week for 2 hours over a 12-week period.	*N* = 8 Age = 9–13	Qualitative, multiple case study	Unclear	Behavior change, views of self, views on health	*Efficacy:* Results discussed feelings of self-esteem and self-worth, accountability and responsibility, connection to community, sense of belonging, knowledge and acquisition of health and life skills, application of skills, and planning and recognizing one’s influence on self and others.	Lack of methodological coherence	Age and developmental ranges of participants; lack of philosophical underpinnings, theory, and specific analytical procedures
Fuller et al. (2013)	Mentor-based program that incorporated sport, physical activity, nutrition, and life skills. Sessions were once a week for 2 hours over 2 12-week periods.	*N* = 8 Age = 10–14	Qualitative, methodology not explicitly reported	Deductive analysis	Program evaluation	*Process:* Results discussed reasons for initial participation (curiosity, excitement, opportunity to play sports) and reasons for continued participation (fun, field trips, novelty and exposure, stayed out of trouble). *Efficacy:* Results also discussed positive developmental outcomes (confidence, connection, character, contribution).	Coherence between theory, method, and analysis	Three of 8 participants were brothers; lack of philosophical underpinning; deductive analytic procedures

While 61 articles are listed in this table, 5 studies were presented in multiple publications (e.g., dissertation and peer-reviewed article, full report and brief report); thus, these were considered duplicate documents

## Results

### Summer Sport and Life Skills Camps

#### Study quality

Summer Sport and Life Skills Camps (4–5 weeks in length) grounded in principles of positive youth development (e.g., caring climate, support for autonomy and competence) and sponsored by the National Youth Sport Program or universities were assessed in 11 studies (8 quantitative, 2 qualitative, 1 mixed methods). Eight studies with quantitative components were classified as weak evidence, with only McDavid, McDonough, Blankenship, and LeBreton classified as moderate evidence [25]. Most studies were single-group, pre-test post-test designs. Of the 2 qualitative studies and 1 mixed methods study, 1 was assessed as having no methodological coherence, while 2 were assessed as partial methodological coherence [26, 27].

#### Outcomes

Overall, the findings suggest a limited effect of Summer Sport and Life Skills Camps on youth outcomes, with major limitations being short assessment periods and/or a lack of follow-up. In 1 follow-up study, slight increases were reported in assessment scores for those who returned to camp, but the findings did not account for those who did not return [28]. Given that the outcomes assessed could be considered rather stable (e.g., perceptions of competence, hope, self-worth), it is possible that the short intervention and observation phases were not sensitive enough to show possible changes.

One strength of this body of evidence is the use of mediational and moderation analyses to identify key critical factors. For example, establishing a caring climate increased empathic concern [29], and affective self-regulatory efficacy and empathic self-efficacy helped mediate the link between caring climate and social behaviors [30]. Moreover, leader behavior and perceived leader support were important predictors of child-level outcomes and camp participation in subsequent years [28, 31]. These findings imply that ongoing intervention involvement may enhance outcomes. Ulrich-French and McDonough reported that lower BMI, higher self-worth, and higher intervention attendance were also predictors of ongoing engagement [28]. Finally, findings suggested that the camp interventions were most effective for older children [32] and those who entered with higher levels of baseline risk [33]. Qualitative findings suggested that summer camp interventions were safe places where relationships were developed [27]. No physical health data were assessed.

### Teaching Personal and Social Responsibility

#### Study quality

Teaching Personal and Social Responsibility is a professional practice model that has been used for decades by practitioners and scholars, with intentional programming supporting the development of personal and social responsibility in youth. All 17 studies (2 quantitative, 13 qualitative, 2 mixed methods) were designed and conducted by university faculty and/or students. Three of the 4 studies with a quantitative component were judged as having a weak level of evidence, with 1 study [34] rated as strong evidence. Of the 15 studies with a qualitative component, 6 studies were assessed as having no methodological coherence, 8 studies partial methodological coherence, and 1 study full methodological coherence [35]. Common weaknesses of qualitative studies were a lack of philosophical or theoretical basis to the study, an over-reliance on deductive/confirmatory analyses, and an over-reliance on participant perspectives, without triangulation with other agents in the intervention. Strengths of qualitative studies included multiple sources of data.

#### Outcomes

Overall, lower-rated qualitative studies reported that participants experienced some development related to the Teaching Personal and Social Responsibility levels (e.g., respect, effort, leadership), and the higher-rated qualitative study [35] identified life skills learned through the intervention including, but not limited to, these levels (e.g., self-belief, courage, responsibility, self-control, self-direction), although the findings were based on participants’ self-reporting. Critical intervention elements that may have enhanced youth outcomes included quality adult-youth relationships, youth leadership, and a youth-centered task-oriented climate [35–42]. Additionally, sustained intervention engagement was perceived to enhance youth development outcomes [36, 38].

While studies often discussed participants transferring skills learned in the intervention to other life domains, actual evidence of transfer was scarce and inconsistent. Hayden et al. [36] and Cryan and Martinek [43] reported a transfer effect into classroom settings, while Jacobs [44] and Wright et al. [45] reported no group differences for intervention and control participants. Despite the fact that the ultimate goal of the Teaching Personal and Social Responsibility model is to have participants transfer life skills outside of the intervention, the current evidence base does not support this outcome. Findings did indicate the need to design a curriculum with opportunities to practice life skills within and outside of the intervention context [41], which may support life skill transfer.

The 1 study with strong evidence [34] examined an intervention within the context of self-determination theory and goal perspective theory, while targeting moral reasoning. Results showed positive effects on distributive justice reasoning and perceived competence for those in the intervention. Thus, it appears that the Teaching Personal and Social Responsibility model can yield some desired outcomes; however, a more substantive and theoretically based intervention may need to be used in conjunction with this professional practice model to elicit positive changes in youth development outcomes.

### Girls on the Run

#### Study quality

Girls on the Run is a non-profit organization that engages girls in community running interventions designed to build physical, psychological, and social assets. Five published studies and 3 evaluation reports (6 quantitative, 2 mixed methods) were assessed. All 8 studies were rated as weak evidence, with 6 single-group studies and 1 cross-sectional study examining exposure and no exposure to the intervention along with 1 study with a 3-group, quasi-experimental design. For the 2 mixed-methods studies, the qualitative components were assessed as having no methodological coherence.

#### Outcomes

While pre-post data provided consistent evidence of small, but positive effects for self-esteem and body image satisfaction, these findings were not upheld by multiple-group designs. Pettee Gabriel, DeBate, High, and Racine [46] found a group x time interaction for self-reported levels of physical activity, with those not exposed and those newly exposed reporting increases in physical activity, while those previously exposed not reporting physical activity increases. More rigorous research should be conducted to examine how changes in physical activity might mediate mental health and wellness-related outcomes. Overall, findings suggested that initial exposure to Girls on the Run likely has the greatest effect and that there may be a ceiling effect for intervention benefit [47]. Social support and the development of self-worth also appear to be important factors in intervention success [48].

### Playworks

#### Study quality

Playworks is a non-profit organization which places trained coaches in schools serving low-income communities, with a focus on providing opportunities for developmentally appropriate physical activity. Two quantitative studies (published across 4 reports [49–52]), 2 qualitative studies [53, 54], and 1 mixed methods study [55] assessed the Playworks intervention. Two quantitative studies were rated as strong level of evidence, and the mixed methods study by Massey et al. [55] was rated as moderate level of evidence. The 2 qualitative studies [53, 54] and the mixed methods study [55] showed partial coherence, as none discussed their underpinning ontology/epistemology or provided a clear rationale for sampling.

#### Outcomes

Madsen et al. [52] reported that with each additional year of exposure to Playworks, youth reported significantly higher scores in physical activity, school participation, problem solving skills, and goal aspirations. While the effects reported were small on a yearly basis, they were clinically meaningful when considered cumulatively (e.g., approximately 1 SD increases in physical activity, meaningful participation, and problem solving across the analysis period). Similarly, other researchers reported higher levels of objectively measured physical activity; higher levels of teacher-reported safety and inclusion both at recess and in school; lower levels of transition difficulties and bullying behaviors; and higher levels of student-reported positive behavior and attention in the classroom [49–51]. However, these same researchers reported no differences between Playworks and control schools on several variables (e.g., student perceptions of safety at recess and aggressive behavior at school, teacher-reported classroom behavior, positive youth development, academic performance). Finally, Massey and colleagues reported improved classroom behavior over time for those in the Playworks peer leadership training intervention, and both within and between-group improvements in playground conflict and positive playground interactions [55]. Qualitative data from London et al. [53] and Massey et al. [54, 55] documented increased recess quality over time, as well as students’ perceptions of an improved recess experience and their own roles as leaders on the playground. Given the quality of the reported evidence, in conjunction with the findings, data suggest that the Playworks intervention facilitates several positive outcomes aligned with public health goals.

### The First Tee

#### Study quality

The First Tee is a non-profit organization that provides youth with facilities and education interventions designed to foster the development of character and values through golf. Three studies conducted on The First Tee were assessed. One study used a qualitative methodology and was assessed to have partial methodological coherence, while 2 studies used quantitative designs, with 1 rated as weak evidence and the other as moderate evidence.

#### Outcomes

The qualitative study [56] identified various life skills that youth perceived to have developed (e.g., ability to meet/greet others, show respect, control emotions), with findings corroborated by a quantitative longitudinal study [57]. Notably, findings implied significant group differences (when controlling for parent education and socioeconomic status) on 5 of 8 life skill transfer domains and 6 of 8 development outcome domains for those with and without exposure. Longitudinal findings also implied that 3 life skills (i.e., meeting/greeting, appreciating diversity, getting help) increased over time and with increased intervention exposure. Brunelle, Danish, and Forneris’ study was more limited in scope, as it only tested outcomes after a 1-week intensive intervention [58]. Results showed short-term improvements in social responsibility and goal knowledge, although no findings were reported on physical health outcomes.

The First Tee findings suggest several potential moderating variables. First, those who entered the intervention with the lowest life skills scores improved the most [57], suggesting this intervention is effective when targeting the appropriate youth. Additionally, those engaging in community service had more favorable outcomes [58], suggesting a need to engage youth outside of the intervention. Finally, girls reported greater increases in perspective taking, while those who identified as White reported greater increases in social interests [58], suggesting a need to better tailor the intervention and outcomes to the unique needs of males, as well as youth from racially and ethnically diverse backgrounds.

Overall, while limited, the findings suggested a positive effect on life skills development for youth in The First Tee, with those findings strengthened by the inclusion of multiple-group data, longitudinal data, qualitative data, and first-person reports, as well as the identification of various critical features (e.g., baseline risk, gender, race, engagement outside of the intervention). No physical health data were assessed.

### Play It Smart

#### Study quality and outcomes

Play It Smart is a school-based intervention that promotes social and personal development for student-athletes, with approximately 50 schools still implementing the intervention in various forms. Two studies were assessed, with the intervention founder an author on both articles. The quantitative study was rated as weak evidence, while the qualitative study was assessed as having no methodological coherence despite using data from exit interview forms from 1361 youth. Overall, findings partially support positive youth development and academic outcomes for participants in Play It Smart, although an overall lack of methodological processes and procedures limits the interpretation of the findings. Physical health findings were not reported.

### Urban Squash

#### Study quality and outcomes

Two Urban Squash interventions facilitated by non-profit organizations were evaluated. Interventions focused on academic enrichment, sports, mentoring, and community service through after-school and summer programming. Three studies (2 quantitative, 1 qualitative) were assessed. Doctoral dissertations represented the quantitative studies, which were rated as moderate evidence and limited by possible selection bias and high attrition rates. The qualitative study was assessed as having no methodological coherence. Overall, there is inconclusive evidence to suggest that these Urban Squash interventions have a positive impact on academic skills, and physical health findings were not reported.

### Coach Across America

#### Study quality and outcomes

Coach Across America is the flagship intervention of Up2Us Sports, where coaches are trained and supported in their efforts to provide quality sport-based youth development for participants in community-based organizations. Coach Across America was assessed with 2 evaluation reports (1 quantitative, 1 mixed methods). The mixed methods study was rated as moderate level quantitative evidence, with the qualitative component assessed as having no methodological coherence. The quantitative study was rated as weak due to missing data and unclear data collection procedures (e.g., reliability and validity of instruments, blinding). Despite findings suggesting improvements in self-reported physical activity and physical fitness tests and the development of high-impact attributes, the findings should be examined cautiously, given methodological concerns. Better results on youth development outcomes were reported for younger participants and those with higher levels of organizational contact.

### Doc Wayne

#### Study quality and outcomes

Doc Wayne is a non-profit organization that provides sport-based group therapy interventions for at-risk youth, along with facilitating the use of its sport-based therapeutic curriculum in varied settings. Two quantitative studies (1 published, 1 evaluation report) were rated as weak evidence, although results demonstrated positive trends for those participating in a trauma-informed sports league on participant behavior (e.g., internalizing and externalizing behavior) and levels of emotional regulation. No findings were reported on physical health outcomes.

### Sport Hartford

#### Study quality and outcomes

Through Sport Hartford (designed for preadolescent females) and Sport Hartford Boys (designed for preadolescent males), sport and physical activity are used to help participants develop their intellectual, social, and physical competencies. Both interventions are designed and conducted by university faculty and/or students. Three qualitative studies were published. One study was assessed as having no methodological coherence, while the other 2 were assessed as having partial methodological coherence. All studies were limited by low participant numbers. Findings suggest that Sport Hartford shows promise but currently lacks efficacy data, along with findings on physical health outcomes. Broader principles ascertained from these studies suggest that interventions need to maintain a sense of fun, provide unique experiences, help youth develop broader life and community outcomes, and create a positive structure that youth can count on for consistency and stability.

## Discussion

This systematic review of research on sport-based youth development interventions conducted within the U.S. over the past 23 years provides a portrait of the current state of evidence. Specifically, there is limited efficacy data, with the quality of methods and evidence largely classified as weak and incoherent. A number of recommendations are offered from this review. These include the need to: (a) assess intervention quality and fidelity; (b) use multiple groups, incorporate multi-site comparison, and use longitudinal designs; (c) account for confounding variables (e.g., maturation bias, selection bias); and (d) integrate studies across philosophical, theoretical, methodological, and analytical perspectives. Additionally, the norms for reporting research on sport-based youth development interventions must be critically examined and improved. Despite more than 700 organizations operating in the U.S. [59], only 56 studies assessing 10 intervention types met the inclusion criteria for this review, with many of the articles limited in their transparency (e.g., unclear sampling procedures, infrequent null/negative outcomes). Likely reasons include funding concerns (e.g., fear of reporting null/negative outcomes), time limitations, word count limits, and publication bias. This is alarming if we consider that the evidence base is grounded in what is publicly shared. Not only does this limit intervention and research efficacy, but it also means that decisions related to funding and policy may be grounded in research that does not meet high quality standards. Thus, the quality of methods and evidence–and the reporting of these–needs to improve so that intervention efficacy can be assessed systematically to fully appreciate the potential for sport-based youth development to contribute to public health goals, and to more precisely understand its limitations. There are certainly challenges inherent to this, as research in this field is fraught with hurdles and limitations (e.g., respondent burden, degradation of data quality), but this does not change the need for higher quality of methods and evidence which are widely and transparently reported.

Limited efficacy data constrains the conclusions that can be made from this systematic review, with very few higher-quality studies scattered across the 10 reported intervention types. Nevertheless, processes likely to contribute to intervention outcomes were identified, although more rigorous designs are needed to fully examine these. For instance, there is a need to design curricula around predictors of ongoing engagement (e.g., leader behavior, higher self-worth, safe climate), with extended engagement in interventions likely to lead to improved participant outcomes. Additionally, the most appropriate target population for each intervention must be carefully considered, from participant age, gender, and race/ethnicity to life skill scores and baseline risk levels. As for intervention design, the following features were identified as variables influencing youth experiences and outcomes: (a) climate (e.g., safety, sense of caring/support, trust, stability); (b) leadership (e.g., support, adult-youth relationships, training/education); (c) youth engagement (e.g., youth leadership, ability to practice life skills); and (d) activities (e.g., fun, novelty), including engagement outside the intervention itself (e.g., community service, ability to practice life skills, connections to broader life/community outcomes). These and other processes must be rigorously studied in greater depth to understand whether, how, and why sport-based youth development interventions may facilitate certain outcomes and impacts. Additionally, if sport-based youth development interventions are going to contribute to the public health agenda, there is a need for concurrent assessment of the physical, cognitive, affective, social, and lifestyle domains identified by Bailey [13], with a particular focus on physical health. In the 10 intervention types featured in this systematic review, only 3 (i.e., Girls on the Run, Playworks, Coach Across America) directly measured physical activity or physical fitness levels, despite widespread concerns about physical inactivity and overweight/obese classifications in the U.S. [2, 3]. This suggests that sport-based youth development interventions may be prioritizing the cognitive, affective, social, and lifestyle domains, thereby eschewing the physical domain that is inherent to sport-based interventions; this must be addressed in future studies. This knowledge can inform future theory, research, praxis, policy, and funding, along with the broader dialogue surrounding sport-based youth development within the public health agenda.

### Limitations

The limitations begin with the scant methodological descriptions provided in many of the included studies, but extend to publication bias, self-censorship, and the geopolitics of knowledge production in these studies. As is the case with many systematic reviews, the research team could not pool the data for meta-analysis due to heterogeneity. The decision to require a minimum of 2 independent records for an intervention type excluded 25 studies, although only 1 study published in 2002 [60] was rated above weak evidence and no studies were assessed as having full methodological coherence, suggesting this criterion did not overlook higher-quality published research on smaller, newer interventions.

## Conclusions

Returning to the primary research question guiding this systematic review, the quality of evidence reported for sport-based youth development interventions in the U.S. was largely classified as weak and incoherent. This limited efficacy data therefore constrained the conclusions that could be made about the secondary research questions, which related to intervention efficacy in improving public health-related goals and identification of processes which contribute to intervention outcomes.

Thus, the quality of the reviewed evidence base does not yet warrant wide scale implementation of sport-based youth development interventions for public health goals within the U.S., although there is promising research that identifies areas for exploration. Further investigation is needed to elucidate the processes that contribute to the outcomes of sport-based youth development interventions. This includes ideal adult-youth ratios in interventions, effective leader training and education, and optimal intervention size and dosage, along with the recommendations offered above. Additionally, more questions need to be asked about the efficacy of sport-based youth development interventions contributing to public health-related goals, particularly within larger systems (e.g., schools, communities). For example, given that social inequalities and neighborhood characteristics disproportionately affect the health of low-income youth, how should interventions be adapted to best serve this population? Further, what is the social return on investment for sport-based youth development interventions related to public health goals? These questions, and others, must be rigorously and comprehensively studied to appreciate the most effective, efficient approach for sport-based youth development within the public health agenda.

## Additional files

Additional file 1: Prisma checklist, Completed PRISMA checklist to allow methodology to be fully evaluated and utilized. (DOC 69 kb)

Additional file 2: Search strategies, Search strategies used to find studies for the review. (DOCX 27 kb)

Additional file 3: References for Studies in Table 1 (in order of appearance in table), References for Studies in Table 1 (in order of appearance in table). (DOCX 25 kb)

## Abbreviations

U.S.: United States
