# Supplementary material for: Sport-based youth development interventions in the United States: a systematic review
Source: BMC Public Health. 2019 Jan 18;19:89. doi: 10.1186/s12889-019-6387-z (PMC6339434; doi:10.1186/s12889-019-6387-z)
Supplement: Supplementary file 2 — Search strategies, Search strategies used to find studies for the review. (DOCX 27 kb) [file 12889_2019_6387_MOESM2_ESM.docx]

**Appendix B: Search Strategies**

**PsycInfo**

1. child.ti,ab.

2. children.ti,ab.

3. minor.ti,ab.

4. minors.ti,ab.

5. youth.ti,ab.

6. youths.ti,ab.

7. young.ti,ab.

8. youngster?.ti,ab.

9. adolescent?.ti,ab.

10. preadolescent?.ti,ab.

11. girl.ti,ab.

12. girls.ti,ab.

13. boy.ti,ab.

14. boys.ti,ab.

15. teen?.ti,ab.

16. teenage*.ti,ab.

17. student?.ti,ab.

18. juvenile?.ti,ab.

19. kid.ti,ab.

20. kids.ti,ab.

21. school age?.ti,ab.

22. or/1-21

23. exp sports/

24. physical activity/

25. physical fitness/

26. yoga/

27. sport?.ti,ab.

28. extracurricular?.ti,ab.

29. extra curricular?.ti,ab.

30. physical activit*.ti,ab.

31. physical fitness.ti,ab.

32. athlet*.ti,ab.

33. football.ti,ab.

34. soccer.ti,ab.

35. tennis.ti,ab.

36. swimming.ti,ab.

37. running.ti,ab.

38. cycling.ti,ab.

39. basketball.ti,ab.

40. baseball.ti,ab.

41. martial art?.ti,ab.

42. yoga.ti,ab.

43. or/23-42

44. mentor/

45. role models/

46. social change/

47. psychosocial

development/

48. peace*.ti,ab.

49. mentor*.ti,ab.

50. role model?.ti,ab.

51. social change.ti,ab.

52. (sport? adj3

development*).ti,ab.

53. (youth adj3 develop*).ti,ab.

54. social* adj3

develop*).ti,ab.

55. (psychosocial* adj3

develop*).ti,ab.

56. (econom* adj3

develop*).ti,ab.

57. (communit* adj3

develop*).ti,ab.

58. or/44-57

59. sport? for

development.ti,ab.

60. sport? for youth.ti,ab.

61. National Youth Sport

Program.ti,ab.

62. 59 or 60 or 61

63. 22 and 43 and 58

64. 62 or 63

65. limit 64 to yr="1995-2018"

66. (animal not human).po.

67. 65 not 66

**Embase**

1. exp child/

2. exp adolescent/

3. juvenile/

4. child.ti,ab.

5. children.ti,ab.

6. minor.ti,ab.

7. minors.ti,ab.

8. youth.ti,ab.

9. youths.ti,ab.

10. young.ti,ab.

11. youngster?.ti,ab.

12. adolescent?.ti,ab.

13. preadolescent?.ti,ab.

14. girl.ti,ab.

15. girls.ti,ab.

16. boy.ti,ab.

17. boys.ti,ab.

18. teen?.ti,ab.

19. teenage*.ti,ab.

20. student?.ti,ab.

21. juvenile?.ti,ab.

22. kid.ti,ab.

23. kids.ti,ab.

24. school age?.ti,ab.

25. or/1-24

26. exp sport/

27. exp physical activity/

28. physical education/

29. fitness/

30. exercise/

31. sport?.ti,ab.

32. extracurricular?.ti,ab.

33. extra curricular?.ti,ab.

34. physical activit*.ti,ab.

35. physical fitness.ti,ab.

36. athlet*.ti,ab.

37. football.ti,ab.

38. soccer.ti,ab.

39. tennis.ti,ab.

40. swimming.ti,ab.

41. running.ti,ab.

42. cycling.ti,ab.

43. basketball.ti,ab.

44. baseball.ti,ab.

45. martial art?.ti,ab.

46. yoga.ti,ab.

47. or/26-46

48. social change/

49. social evolution/

50. peace*.ti,ab.

51. mentor*.ti,ab.

52. role model?.ti,ab.

53. social change.ti,ab.

54. (sport? adj3 development*).ti,ab.

55. (youth adj3 develop*).ti,ab.

56. (social* adj3 develop*).ti,ab.

57. (psychosocial* adj3 develop*).ti,ab.

58. (econom* adj3 develop*).ti,ab.

59. (communit* adj3 develop*).ti,ab.

60. or/48-59

61. sport? for development.ti,ab.

62. sport? for youth.ti,ab.

63. National Youth Sport Program.ti,ab.

64. 61 or 62 or 63

65. 25 and 47 and 60

66. 64 or 65

67. (exp animal/ or nonhuman/) not exp human/

68. 66 not 67

69. conference abstract.pt.

70. 68 not 69

71. limit 70 to yr="1995 - 2017"

**SPORTDiscus**

| **S12** | S1 OR (S4 AND S7 AND S10)  Limiters - Published Date: 19950101-20171231 |
| --- | --- |
| **S11** | S1 OR (S4 AND S7 AND S10) |
| **S10** | S8 OR S9 |
| **S9** | (((DE "CHILDREN" OR DE "AIDS (Disease) & children" OR DE "BOYS" OR DE "CHILD acrobats" OR DE "CHILD circus performers" OR DE "CHILD dancers" OR DE "CHILD development" OR DE "DANCE for children" OR DE "DEAFBLIND children" OR DE "GIRLS" OR DE "OUTDOOR recreation for children" OR DE "OVERWEIGHT children" OR DE "SCHOOL children" OR DE "SELF-defense for children" OR DE "VIDEO games & children")) OR (DE "TEENAGERS")) OR (DE "YOUTH" OR DE "SCOUTS (Youth organization members)" OR DE "TEENAGERS" OR DE "YOUNG adults" OR DE "YOUTH -- Recreation") |
| **S8** | (TI child OR AB child) OR (TI children OR AB children) OR (TI minor OR AB minor) OR (TI minors OR AB minors) OR (TI youth OR AB youth) OR (TI youths OR AB youths) OR (TI young OR AB young) OR (TI youngster# OR AB youngster#) OR (TI adolescent# OR AB adolescent#) OR (TI preadolescent# OR AB preadolescent#) OR (TI girl OR AB girl) OR (TI girls OR AB girls) OR (TI boy OR AB boy) OR (TI boys OR AB boys) OR (TI teen# OR AB teen#) OR (TI teenage* OR AB teenage*) OR (TI student# OR AB student#) OR (TI juvenile# OR AB juvenile#) OR (TI kid OR AB kid) OR (TI kids OR AB kids) OR (TI "school age#" OR AB "school age#") |
| **S7** | S5 OR S6 |
| **S6** | ((DE "SPORTS -- Sociological aspects") OR (DE "SPORT for all")) OR (DE "EXERCISE for children" OR DE "TAI chi for children" OR DE "YOGA for children") |
| **S5** | (TI sport# OR AB sport#) OR (TI extracurricular# OR AB extracurricular#) OR (TI "extra curricular#" OR AB "extra curricular#") OR (TI "physical activit*" OR AB "physical activit*") OR (TI "physical fitness" OR AB "physical fitness") OR (TI athlet* OR AB athlet*) OR (TI football OR AB football) OR (TI soccer OR AB soccer) OR (TI tennis OR AB tennis) OR (TI swimming OR AB swimming) OR (TI running OR AB running) OR (TI basketball OR AB basketball) OR (TI baseball OR AB baseball) OR (TI cycling OR AB cycling) OR (TI "martial art#" OR AB "martial art#") OR (TI yoga OR AB yoga) |
| **S4** | S2 OR S3 |
| **S3** | ((DE "MENTORING") OR (DE "ROLE models")) OR (DE "SPORTS for youth") |
| **S2** | (TI mentor* OR AB mentor*) OR (TI "role model#" OR AB "role model#") OR (TI "social change" OR AB "social change") OR (TI peace* OR AB peace*) OR (TI sport# N3 development* OR AB sport# N3 development*) OR (TI youth N3 develop* OR AB youth N3 develop*) OR (TI social* N3 develop* OR AB social* N3 develop*) OR (TI psychosocial* N3 develop* OR AB psychosocial* N3 develop*) OR (TI econom* N3 develop* OR AB econom* N3 develop*) OR (TI communit* N3 develop* OR AB communit* N3 develop*) |
| **S1** | (TI "sport# for youth" OR AB "sport# for youth") OR (TI "sport# for development" OR AB "sport# for development") OR (TI "national youth sport program" OR AB "national youth sport program") |

**Education Source**

| **S13** | S10 OR S11  Limiters - Published Date: 19950101-20171231 |
| --- | --- |
| **S12** | S10 OR S11 |
| **S11** | S3 AND S6 AND S9 |
| **S10** | (DE "Physical education -- Moral & ethical aspects") OR (TI "sport# for youth" OR AB "sport# for youth") OR (TI "sport# for development" OR AB "sport# for development") OR (TI "national youth sport program" OR AB "national youth sport program") |
| **S9** | S7 OR S8 |
| **S8** | (TI mentor* OR AB mentor*) OR (TI "role model#" OR AB "role model#") OR (TI "social change" OR AB "social change") OR (TI peace* OR AB peace*) OR (TI sport# N3 development* OR AB sport# N3 development*) OR (TI youth N3 develop* OR AB youth N3 develop*) OR (TI social* N3 develop* OR AB social* N3 develop*) OR (TI psychosocial* N3 develop* OR AB psychosocial* N3 develop*) OR (TI econom* N3 develop* OR AB econom* N3 develop*) OR (TI communit* N3 develop* OR AB communit* N3 develop*) |
| **S7** | (((DE "Personality development") OR (DE "Social development")) OR (DE "Economic development" OR DE "Community development")) OR (DE "Moral education" OR DE "Bioethics -- Study & teaching" OR DE "Humane education" OR DE "Jewish religious education" OR DE "Medical ethics -- Study & teaching" OR DE "Moral education (Early childhood)" OR DE "Moral education (Elementary)" OR DE "Moral education (Higher)" OR DE "Moral education (Middle school)" OR DE "Moral education (Preschool)" OR DE "Moral education (Primary)" OR DE "Moral education (Secondary)" OR DE "Values (Ethics) -- Study & teaching" OR DE "Values clarification") |
| **S6** | S4 OR S5 |
| **S5** | (TI sport# OR AB sport#) OR (TI extracurricular# OR AB extracurricular#) OR (TI "extra curricular#" OR AB "extra curricular#") OR (TI "physical activit*" OR AB "physical activit*") OR (TI "physical fitness" OR AB "physical fitness") OR (TI athlet* OR AB athlet*) OR (TI football OR AB football) OR (TI soccer OR AB soccer) OR (TI tennis OR AB tennis) OR (TI swimming OR AB swimming) OR (TI running OR AB running) OR (TI basketball OR AB basketball) OR (TI baseball OR AB baseball) OR (TI cycling OR AB cycling) OR (TI "martial art#" OR AB "martial art#") OR (TI yoga OR AB yoga) |
| **S4** | (DE "After school sports") OR (DE "Athletics" OR DE "Athletic tryouts" OR DE "College sports" OR DE "Track & field") |
| **S3** | S1 OR S2 |
| **S2** | (TI child OR AB child) OR (TI children OR AB children) OR (TI minor OR AB minor) OR (TI minors OR AB minors) OR (TI youth OR AB youth) OR (TI youths OR AB youths) OR (TI young OR AB young) OR (TI youngster# OR AB youngster#) OR (TI adolescent# OR AB adolescent#) OR (TI preadolescent# OR AB preadolescent#) OR (TI girl OR AB girl) OR (TI girls OR AB girls) OR (TI boy OR AB boy) OR (TI boys OR AB boys) OR (TI teen# OR AB teen#) OR (TI teenage* OR AB teenage*) OR (TI student# OR AB student#) OR (TI juvenile# OR AB juvenile#) OR (TI kid OR AB kid) OR (TI kids OR AB kids) OR (TI "school age#" OR AB "school age#") |
| **S1** | ((DE "Children" OR DE "Abused children" OR DE "Adopted children" OR DE "Birth order" OR DE "Black children" OR DE "Boys" OR DE "Child development" OR DE "Children as teachers" OR DE "Children of attention-deficit-disordered parents" OR DE "Children of divorced parents" OR DE "Children of foreign workers" OR DE "Children of immigrants" OR DE "Children of migrant laborers" OR DE "Children of minorities" OR DE "Children of older parents" OR DE "Children of parents with disabilities" OR DE "Children of school principals" OR DE "Children of single parents" OR DE "Children of teenage mothers" OR DE "Children of the rich" OR DE "Children of unmarried parents" OR DE "Children of working parents" OR DE "Children's television programs" OR DE "City children" OR DE "Exceptional children" OR DE "Foster children" OR DE "Girls" OR DE "Grandchildren" OR DE "Handicraft for children" OR DE "Homeless children" OR DE "Mentally ill children" OR DE "Playmates" OR DE "Poor children" OR DE "Preschool children" OR DE "Problem children" OR DE "Refugee children" OR DE "Religious education of children" OR DE "School children") OR (DE "Youth" OR DE "At-risk youth" OR DE "Bisexual youth" OR DE "Black youth" OR DE "Juvenile delinquents" OR DE "LGBT youth" OR DE "Mentally ill youth" OR DE "Minority youth" OR DE "Problem youth" OR DE "Religious education of young people" OR DE "School dropouts" OR DE "Teenagers" OR DE "Urban youth" OR DE "Young adults" OR DE "Youth with disabilities")) OR (DE "Students" OR DE "Adult education students" OR DE "Adult students" OR DE "Advanced students" OR DE "African students" OR DE "Agricultural students" OR DE "American students" OR DE "Animation students" OR DE "Anthropology students" OR DE "Appropriate education" OR DE "Arab students" OR DE "Arabic-speaking students" OR DE "Art students" OR DE "Arts students" OR DE "Asian students" OR DE "At-risk students" OR DE "Australian students" OR DE "Bhutanese students" OR DE "Bilingual students" OR DE "Bisexual students" OR DE "Black students" OR DE "Boarding school students" OR DE "British students" OR DE "Business students" OR DE "Chinese-speaking students" OR DE "Christian students" OR DE "Church work with students" OR DE "Classes (Groups of students)" OR DE "College students" OR DE "Culinary students" OR DE "Dental students" OR DE "Design students" OR DE "Distance education students" OR DE "Dutch students" OR DE "Education students" OR DE "Engineering students" OR DE "European students" OR DE "Evening & continuation school students" OR DE "Filipino students" OR DE "Foreign students" OR DE "French-speaking students" OR DE "Full-time students" OR DE "Gay students" OR DE "German-speaking students" OR DE "Handicraft students" OR DE "Health occupations students" OR DE "History students" OR DE "Home economics students" OR DE "Homeless students" OR DE "Immigrant students" OR DE "Information science students" OR DE "Interior decoration students" OR DE "Inuit students" OR DE "Japanese-speaking students" OR DE "Jewish students" OR DE "Journalism students" OR DE "Korean-speaking students" OR DE "LGBT students" OR DE "Landscape architecture students" OR DE "Law students" OR DE "Lesbian students" OR DE "Libraries & students" OR DE "Library school students" OR DE "Literature students" OR DE "Low-income students" OR DE "Married students" OR DE "Mathematics students" OR DE "Medical students" OR DE "Middle school students" OR DE "Minority students" OR DE "Modern language students" OR DE "Moldovan students" OR DE "Music students" OR DE "Muslim students" OR DE "Native American students" OR DE "Non-matriculated students" OR DE "Nutrition students" OR DE "Older students" OR DE "Osteopathic students" OR DE "Out-of-state students" OR DE "Part-time students" OR DE "Political science students" OR DE "Pregnant students" OR DE "Preparatory school students" OR DE "Private school students" OR DE "Public speaking students" OR DE "Re-entry students" OR DE "School children" OR DE "School dropouts" OR DE "Science students" OR DE "Secondary school students" OR DE "Self-efficacy in students" OR DE "Self-supporting students" OR DE "Seminarians" OR DE "Sixth form students" OR DE "Social science students" OR DE "Social work students" OR DE "Sociology students" OR DE "Spanish-speaking students" OR DE "Special needs students" OR DE "Stopouts" OR DE "Student pacifists" OR DE "Student problems" OR DE "Student protesters" OR DE "Student volunteers" OR DE "Students & war" OR DE "Students -- Services for" OR DE "Students as military personnel" OR DE "Students with disabilities" OR DE "Technology students" OR DE "Transfer of students" OR DE "Transfer students" OR DE "Transgender students" OR DE "Transsexual students" OR DE "Underachievers" OR DE "Vocational school students") |

**Scopus**

((TITLE-ABS-KEY(child OR minors OR youth OR young OR youngster OR adolescent OR preadolescent OR girl OR boy OR teen OR teenage OR student OR juvenile OR kid OR "school age") AND TITLE-ABS-KEY(sport OR extracurricular OR "extra curricular" OR "physical activity" OR "physical fitness" OR athlet* OR football OR soccer OR tennis OR swimming OR running OR basketball OR baseball OR cycling OR "martial art" OR yoga) AND TITLE-ABS-KEY(mentor* OR "role model" OR "social change" OR peace* OR "for development" OR "for developmental" OR "in development" OR "in developmental" OR (sport W/3 development*) OR (youth W/3 develop*) OR (social W/3 develop*) OR (psychosocial W/3 develop*) OR (communit* W/3 develop*) OR (econom* W/3 develop*))) OR (TITLE-ABS-KEY("sport for youth" OR "sport for development" OR "national youth sport program"))) AND (PUBYEAR > 1994 AND LIMIT-TO(SRCTYPE,"j" ) )

**Web of Science**

|  | 1995 – 2016  Indexes=SCI-EXPANDED, SSCI, A&HCI |
| --- | --- |
| #6 | #5 OR #4  DocType=All document types; Language=All languages; |
| #5 | #1 AND #2 AND #3  DocType=All document types; Language=All languages; |
| #4 | TOPIC: ("sport for youth” OR "sport for development” OR "sports for youth” OR "sports for development” OR "national youth sport program”)  DocType=All document types; Language=All languages; |
| #3 | TOPIC: (mentor* OR "role model" OR "role models" OR "social change" OR peace* OR "for development" OR "for developmental" OR “in development” OR “in developmental” OR (sport NEAR/3 development*) OR (youth NEAR/3 develop*) OR (social* NEAR/3 develop*) OR (psychosocial* NEAR/3 develop*) OR (communit* NEAR/3 develop*) OR (econom* NEAR/3 develop*))  DocType=All document types; Language=All languages; |
| #2 | TOPIC: (sport OR extracurricular$ OR "extra curricular" OR "extra curriculars" OR "physical activity" OR "physical activities" OR "physical fitness" OR athlet* OR football OR soccer OR tennis OR swimming OR running OR basketball OR baseball OR cycling OR "martial art" OR "martial arts" OR yoga)  DocType=All document types; Language=All languages; |
| #1 | TOPIC: (child OR children OR minor OR minors OR youth OR youths OR young OR youngster$ OR adolescent$ OR preadolescent$ OR girl OR girls OR boy OR boys OR teen$ OR teenage* OR student$ OR juvenile$ OR kid OR kids OR "school age" OR "school aged")  DocType=All document types; Language=All languages; |
