# Supplementary material for: Sport-based youth development interventions in the United States: a systematic review
Source: BMC Public Health. 2019 Jan 18;19:89. doi: 10.1186/s12889-019-6387-z (PMC6339434; doi:10.1186/s12889-019-6387-z)
Supplement: Supplementary file 3 — References for Studies in Table 1 (in order of appearance in table), References for Studies in Table 1 (in order of appearance in table). (DOCX 25 kb) [file 12889_2019_6387_MOESM3_ESM.docx]

**Appendix C: References for Studies in Table 1 (in order of appearance in table)**

Anderson-Butcher D, Iachini A, Riley A, Wade-Mdivanian R, Davis J, Amorose AJ. Exploring the impact of a summer sport-based youth development program. Eval Program Plann. 2013;37:64-69.

Anderson-Butcher D, Riley A, Amorose A, Iachini A, Wade-Mdivanian R. Maximizing youth experiences in community sport settings: the design and impact of the LiFE Sports Camp. Journal of Sport Management. 2014;28(2):236-249.

Gano-Overway LA, Newton M, Magyar TM, Fry MD, Kim MS, Guivernau MR. Influence of caring youth sport contexts on efficacy-related beliefs and social behaviors. Dev Psychol. 2009;45(2):329-340.

McDavid L, McDonough MH, Smith AL. An empirical evaluation of two theoretically-based hypotheses on the directional association between self-worth and hope. J Adolesc. 2015;41:25-30.

McDavid L, McDonough MH, Blankenship BT, LeBreton JM. A test of basic psychological needs theory in a physical-activity-based program for underserved youth. J Sport Exerc Psychol. 2017;39(1):29-42.

Newton M, Watson DL, Gano-Overway L, Fry M, Kim M-S, Magyar M. The role of a caring-based intervention in a physical activity setting. The Urban Review. 2007;39(3):281-299.

Riciputi S, McDonough MH, Ullrich-French S. Participant perceptions of character concepts in a physical activity-based positive youth development program. J Sport Exerc Psychol. 2016;38(5):481-492.

Riley A, Anderson-Butcher D. Participation in a summer sport-based youth development program for disadvantaged youth: getting the parent perspective. Child Youth Serv Rev. 2012;34(7):1367-1377.

Riley A, Anderson-Butcher D, Logan J, Newman TJ, Davis J. Staff practices and social skill outcomes in a sport-based youth program. J Appl Sport Psychol. 2017;29(1):59-74.

Riley AB. Staff practices and youth outcomes in a summer sport-based positive youth development program [doctoral dissertation]. Columbus, OH, The Ohio State University; 2013.

Ullrich-French S, McDonough M. Correlates of long-term participation in a physical activity-based positive youth development program for low-income youth: sustained involvement and psychosocial outcomes. J Adolesc. 2013;36(2):279-288.

Ullrich-French S, McDonough MH, Smith AL. Social connection and psychological outcomes in a physical activity-based youth development setting. Res Q Exerc Sport. 2012;83(3):431-441.

Cryan M, Martinek T. Youth sport development through soccer: an evaluation of an after-school program using the TPSR model. Physical Educator. 2017. 2017;74(1).

Hayden LA, Baltzell A, Kilty K, McCarthy J. Developing responsibility using physical activity: a case study of Team Support. Ágora for Physical Education and Sport. 2012;14(2):264-281.

Hayden LA. The power of a caring climate: assessing the fidelity of team support to Hellison’s responsibility model and student-athletes perceived outcomes of participating in team support [doctoral dissertation]. Boston, MA, Boston University; 2010.

Jacobs JM. What is learned and does it transfer? A survey of physical education students’ perceptions on transfer of life skills [doctoral dissertation]. Paper 2 of 3: What is learned and does it transfer? A survey of physical education students' perceptions on transfer of life skills. DeKalb, IL, Northern Illinois University; 2016.

Jacobs JM. What is learned and does it transfer? A survey of physical education students’ perceptions on transfer of life skills [doctoral dissertation]. Paper 3 of 3: Youth perceptions of the transfer of life skills in a sport-based youth development program. DeKalb, IL, Northern Illinois University; 2016.

Martinek T, Schilling T, Hellison D. The development of compassionate and caring leadership among adolescents. Phys Educ Sport Pedagogy. 2006;11(2):141-157.

Melendez A, Martinek T. Life after Project Effort: applying values acquired in a responsibility-based physical activity program. Revista Internacional de Ciencias del Deporte. 2015;11(41):259-280.

Miller SC. A program to promote the sociomoral growth of at-risk youth (doctoral dissertation; order no. 9827042) available from Proquest dissertations & theses global (304344644). Berkeley, CA, University of California at Berkeley; 1997.

Schilling T, Martinek T, Carson S. Youth leaders' perceptions of commitment to a responsibility-based physical activity program. Res Q Exerc Sport. 2007;78(2):48-60.

Walsh D. Helping youth in underserved communities envision possible futures: an extension of the Teaching Personal and Social Responsibility model. Res Q Exerc Sport. 2008;79(2):209-221.

Walsh DS, Ozaeta J, Wright PM. Transference of responsibility model goals to the school environment: exploring the impact of a coaching club program. Phys Educ Sport Pedagogy. 2010;15(1):15-28.

Walsh DS, Veri MJ, Scobie D. Impact of the Kinesiology Career Club: a TPSR-based possible futures program for youth in underserved communities. Ágora for Physical Education and Sport. 2012;14(2):213-229.

Walsh DS, Veri MJ, Willard JJ. Kinesiology Career Club: undergraduate student mentors' perspectives on a physical activity-based Teaching Personal and Social Responsibility program. Physical Educator. 2015;72(2):317-339.

Whitley MA, Coble C, Jewell GS. Evaluation of a sport-based youth development programme for refugees. Leisure/Loisir. 2016;40(2):175-199.

Whitley MA, Massey WV, Farrell K. A programme evaluation of ‘Exploring Our Strengths and Our Future’: making sport relevant to the educational, social, and emotional needs of youth. J Sport Dev. 2017;5(9):21-35.

Wright PM, Burton S. Implementation and outcomes of a responsibility-based physical activity program integrated into an intact high school physical education class. J Teach Phys Educ. 2008;27(2):138-154.

Wright PM, Li W, Ding S, Pickering M. Integrating a personal and social responsibility program into a wellness course for urban high school students: assessing implementation and educational outcomes. Sport Educ Soc. 2010;15(3):277-298.

Wright PM, Dyson B, Moten T. Exploring the individualized experiences of participants in a responsibility-based youth development program. Ágora for Physical Education and Sport. 2012;14(2):248-263.

Beller JM. Positive youth development programs and high school girls’ psychosocial behaviors [doctoral dissertation]. Phoenix, AZ, Grand Canyon University; 2013.

DeBate RD. Girls on the Run international evaluation report. 2002; https://www.girlsontherun.org/assets/docs/2006_Evaluation.pdf.

DeBate RD, Delmar CE. Girls on the Run international formative evaluation report. 2006; https://www.girlsontherun.org/assets/docs/2006_Evaluation.pdf.

DeBate RD, Otero-Fisher KA. Girls on the Run international formative evaluation report. 2005; https://www.girlsontherun.org/assets/docs/2005_Evaluation.pdf.

DeBate RD, Pettee Gabriel K, Zwald M, Huberty J, Zhang Y. Changes in psychosocial factors and physical activity frequency among third- to eighth-grade girls who participated in a developmentally focused youth sport program: a preliminary study. J Sch Health. 2009;79(10):474-484.

Pettee Gabriel KK, DeBate RD, High RR, Racine EF. Girls on the Run: a quasi-experimental evaluation of a developmentally focused youth sport program. J Phys Act Health. 2011;8 Suppl 2:S285-294.

Rauscher L, Kauer K, Wilson BDM. The healthy body paradox: organizational and interactional influences on preadolescent girls’ body image in Los Angeles. Gender & Society. 2013;27(2):208-230.

Waldron JJ. Influence of involvement in the Girls on Track program on early adolescent girls' self-perceptions. Res Q Exerc Sport. 2007;78(5):520-530.

Beyler N, Bleeker M, James-Burdumy A, et al. Findings from an experimental evaluation of Playworks: effects on play, physical activity and recess. Report submitted to the Robert Wood Johnson Foundation. Princeton, NJ: Mathematica Policy Research; May 2013.

Fortson J, James-Burdumy S, Bleeker M, et al. Impact and implementation findings from an experimental evaluation of Playworks: effects on school climate, academic learning, student social skills and behavior. Report submitted to the Robert Wood Johnson Foundation. Princeton, NJ: Mathematica Policy Research; May 2013.

London RACS, Stokes-Guinan K. Playworks implementation in 17 schools nationwide. Report submitted to the Robert Wood Johnson Foundation. Princeton, NJ: Mathematica Policy Research; February 2013.

London RA, Westrich L, Stokes-Guinan K, McLaughlin M. Playing fair: The contribution of high-functioning recess to overall school climate in low-income elementary schools. J Sch Health. 2015;85(1):53-60.

Madsen KA, Hicks K, Thompson H. Physical activity and positive youth development: impact of a school-based program. J Sch Health. 2011;81(8):462-470.

Massey WV, Stellino MB, Holliday M, et al. The impact of a multi-component physical activity programme in low-income elementary schools. Health Educ J. 2017;76(5):517-530.

Massey WV, Stellino MB, Wilkison M, Whitley M. The impact of a recess-based leadership program on urban elementary school students. J Appl Sport Psychol. 2018;30(1):45-63.

Brunelle J, Danish SJ, Forneris T. The impact of a sport-based life skill program on adolescent prosocial values. Appl Dev Sci. 2007;11(1):43-55.

Weiss MR, Stuntz CP, Bhalla JA, Bolter ND, Price MS. ‘More than a game’: impact of The First Tee life skills programme on positive youth development: project introduction and year 1 findings. Qualitative Research in Sport, Exercise and Health. 2013;5(2):214-244.

Weiss MR, Bolter ND, Kipp LE. Evaluation of The First Tee in promoting positive youth development: group comparisons and longitudinal trends. Res Q Exerc Sport. 2016;87(3):271-283.

Petitpas AJ, Van Raalte JL, Cornelius AE, Presbrey J. A life skills development program for high school student-athletes. J Prim Prev. 2004;24(3):325-334.

VanGorden K, Cornelius AE, Petitpas AJ. Looking back: reflections from Play It Smart graduates. Hellenic Journal of Psychology. 2010;7:335-349.

Green HK. The impact of an academic sports-mentoring afterschool program on academic outcomes in at-risk youth [doctoral dissertation]. Philadelphia, PA, Drexel University; 2010.

Hemphill MA, Richards KAR. Without the academic part, it wouldn’t be squash”: youth development in an urban squash program. J Teach Phys Educ. 2016;35(3):263-276.

Hill AB. The impact of an academic sports mentoring program on academic and social variables: A comparison [doctoral dissertation]. Philadelphia, PA, Drexel University; 2012.

Louisiana Public Health Institute. Up2Us sports evaluation report Coach Across America programming in the New Orleans area. New Orleans, LA: Author;2016a.

Louisiana Public Health Institute. Coaching for impact: an evaluation of Up2Us Sports conducted by the Louisiana Public Health Institute. New Orleans, LA: Author;2016b.

Windham A, Basen R, Nguyen QN, Mitchell D. Evaluation of the Coach Across America sports-based youth development program: final report. Rockville, MD: American Institutes for Research;2014.

D’Andrea W, Bergholz L, Fortunato A, Spinazzola J. Play to the whistle: a pilot investigation of a sports-based intervention for traumatized girls in residential treatment. J Fam Violence. 2013;28(7):739-749.

EdgeworkConsulting. Doc Wayne program evaluation. n.d.

Bruening JE, Clark BS, Mudrick M. Sport-based youth development in practice: the long-term impacts of an urban after-school program for girls. Journal of Park and Recreation Administration. 2015;33(2).

Bruening JE, Dover KM, Clark BS. Preadolescent female development through sport and physical activity: a case study of an urban after-school program. Res Q Exerc Sport. 2009;80(1):87-101.

Fuller RD, Percy VE, Bruening JE, Cotrufo RJ. Positive youth development: minority male participation in a sport-based afterschool program in an urban environment. Res Q Exerc Sport. 2013;84(4):469-482.
